# Ketogenic therapy in childhood and adolescence: recommendations of the Brazilian experts group

**DOI:** 10.1055/s-0043-1768676

**Published:** 2023-06-28

**Authors:** Letícia Pereira de Brito Sampaio, Adélia Maria de Miranda Henriques-Souza, Katia Lin, Lenycia de Cassya Lopes Neri, Luciana Midori Inuzuka, Ludmila Inácio de Lima Uchôa, Marcela Marques de Oliveira Gregório, Laura Maria Guilhoto, Maria Augusta Montenegro, Mariana Lunardi, Marielza Veiga, Patricia Azevedo de Lima, Vera Braatz

**Affiliations:** 1 Universidade de São Paulo, Faculdade de Medicina, Hospital das Clínicas, Instituto da Criança e Adolescente, São Paulo SP, Brazil.; 2 Instituto de Medicina Integral Professor Fernando Figueira, Centro de Terapias Cetogênicas do IMIP, Recife PE, Brazil.; 3 Universidade Federal de Santa Catarina, Departamento de Medicina Interna, Divisão de Neurologia, Florianópolis SC, Brazil.; 4 Hospital Sirio Libanês, Departamento de Neurofisiologia, São Paulo SP, Brazil.; 5 Hospital Materno-Infantil de Brasília, Brasília DF, Brazil.; 6 Universidade Federal de São Paulo, Departamento de Neurologia, São Paulo, Brazil.; 7 Universidade Estadual de Campinas, Departamento de Neurologia, São Paulo SP, Brazil.; 8 Universidade Federal de Santa Catarina, Programa de Pós-Graduação em Ciências Médicas, Florianópolis SC, Brazil.; 9 Hospital Universitário Professor Edgard Santos, Salvador BA, Brazil.; 10 Universidade Anhanguera, São Paulo SP, Brazil.; 11 Universidade da Região de Joinville, Departamento de Medicina, Divisão de Neurologia, Joinville SC, Brazil.; 12 Universidade de São Paulo, Hospital Universitário, Divisão de Clínica Pediátrica, São Paulo SP, Brazil.

**Keywords:** Diet, Ketogenic, Drug Resistant Epilepsy, Neurology, Brain Diseases, Dieta Cetogênica, Epilepsia Resistente a Medicamentos, Neurologia, Encefalopatias

## Abstract

Ketogenic dietary therapies (KDTs) are a safe and effective treatment for pharmacoresistant epilepsy in children. There are four principal types of KDTs: the classic KD, the modified Atkins diet (MAD), the medium-chain triglyceride (MCT) diet, and the low glycemic index diet (LGID). The International Ketogenic Diet Study Group recommends managing KDTs in children with epilepsy. However, there are no guidelines that address the specific needs of the Brazilian population. Thus, the Brazilian Child Neurology Association elaborated on these recommendations with the goal of stimulating and expanding the use of the KD in Brazil.

## Resumo

As terapias dietéticas cetogênicas (TDC) são um tratamento seguro e eficaz para epilepsia farmacorresistente em crianças. Existem quatro tipos principais de TDCs: a dieta cetogênica (DC) clássica, a dieta de Atkins modificada (DAM), a dieta de triglicerídeos de cadeia média (DTCM) e a dieta de baixo índice glicêmico (DBIG). O Grupo Internacional de Estudos de Dietas Cetogênicas (International Ketogenic Diet Study Group) propõe recomendações para o manejo da DC em crianças com epilepsia. No entanto, faltam diretrizes que contemplem as necessidades específicas da população brasileira. Assim, a Associação Brasileira de Neurologia Infantil elaborou essas recomendações com o objetivo de estimular e expandir o uso da DC no Brasil.

## INTRODUCTION

 Ketogenic dietary therapies (KDTs) are a well-established non-pharmacological treatment for pharmacoresistant epilepsy in children, adolescents, and adults. 1 The International League Against Epilepsy (ILAE) defines pharmacoresistant epilepsy as the failure of adequate trials of two tolerated, appropriately chosen, and used anti-seizure medication (ASM) schedules, whether as monotherapy or in combination, to achieve seizure freedom. 2

 Currently, there are four main KDTs: the classic KD (and their adapted forms), the modified Atkins diet (MAD), the medium-chain triglyceride diet (MCT), and the low glycemic index diet treatment (LGIT). 3 4 5 6 The KD is rich in fat (71–90% energy), low in carbohydrates (5–19% energy), and provides adequate amounts of protein to support nutritional status. 7

 We identified 8 randomized controlled trials that evaluated the efficacy and safety of KDTs in children with pharmacoresistant epilepsy, leading to their recognition as a scientifically valid treatment. 3 4 8 9 10 11 12 13 A systematic review including 711 children with pharmacoresistant epilepsy (4 months to 18 years) demonstrated that the KDT was more effective than the usual care in achieving seizure freedom and seizure reduction. 14 Additionally, a meta-analysis of uncontrolled studies estimated that 59% of infants (≤ 2 years old) with pharmacoresistant epilepsy achieved ≥ 50% seizure reduction, and 33% became seizure free. 15 These data suggest that KDTs could be effective against pharmacoresistant epilepsy in children. 14 15

 In 2009, the International Ketogenic Diet Study Group published a consensus to provide practical recommendations for managing the KDT in children with epilepsy. 16 In 2018, Kossoff et al. presented a revised version of this guideline. 1

So far, there are no guidelines for managing the KDT in children with pharmacoresistant epilepsy in Brazil. Thus, the Brazilian Child Neurology Association elaborated this document with the goal of stimulating and expanding the use of the KD in Brazil.

## METHODS

A group of 13 Brazilian experts from KD centers of different locations in Brazil met virtually on June 24th, 2021, to discuss best practices for managing KDT in children with pharmacoresistant epilepsy in Brazil. The group consisted of 8 child neurologists, 2 neurologists, and 3 dietitians with expertise in KD. The experts were selected based on their knowledge of the subject and their experience managing patients with epilepsy in Brazil. At this meeting, the discussion was focused on determining the main topics to be included in the article.

After the meeting, an independent literature review was conducted to identify the main international references based on the authors' suggestions. The authors discussed and agreed on the topics and references to be included, and a medical writer contributed to the elaboration of this version, reflecting the authors' positions. All authors reviewed all document versions in several steps until a consensus was reached.

## PATIENT SELECTION

 Previous studies confirm that KDT is an effective non-pharmacological treatment for children, infants, adolescents, and adults with pharmacoresistant epilepsy. 1 The randomized controlled studies evaluating the efficacy and safety of these interventions were carried out in individuals aged between 6 months and 18 years. 3 4 8 11 12 17 18 Furthermore, a 2017 case series has shown that KDTs are a safe and effective treatment for epilepsy in children as young as 6 weeks. 19

 According to International guidelines, KDTs should be offered to children with pharmacoresistant epilepsy after a mean of 2.6 (standard deviation [SD]: 0.9) antiseizure ASM failures. 1 Typically, a child neurologist should establish which patients are candidates for treatment after a comprehensive assessment of their medical history, possible limiting risk factors, and contraindications. 20

## INDICATIONS AND CONTRAINDICATIONS

### Indications

 The International Ketogenic Diet Study Group stated that KDTs should be considered early in the epilepsy management of several specific conditions ( Table 1 ). 1

**Table 1:** Conditions in which KDT has shown a 20% improvement in efficacy above the average response (> 50% seizure reduction)

Angelman syndrome
Complex 1 mitochondrial disorders
Dravet syndrome
Epilepsy with myoclonic–atonic seizures (Doose syndrome)
FIRES
Formula-fed (solely) children or infants with pharmacoresistant epilepsy
Glut1DS
Infantile spasms
Ohtahara syndrome
PDD
Super-refractory status epilepticus
Tuberous sclerosis complex

**Abbreviations:** KDT, ketogenic dietary therapy; FIRES, febrile infection-related epilepsy syndrome; Glut1DS, glucose transporter protein 1 deficiency syndrome; PDD, pyruvate dehydrogenase deficiency.

 The treatment of choice for metabolic conditions, such as Glucose transporter type 1 deficiency syndrome (Glut1DS) and Pyruvate dehydrogenase deficiency (PDD), are KDTs. 1 7 In Glut1DS, impaired glucose transport across brain tissue barriers causes seizures. 21 In PDD, pyruvate cannot be metabolized into acetyl-CoA, resulting in lactic acidosis and seizures. 22 Seizures may or may not manifest in both conditions, which present with energy deficit, so KDTs can provide ketones as an alternative energy source for the developing brain. 1

 The International consensus sets specific recommendations for Glut1DS. 1 The classic KD should be used in infants and preschool children; and if well-tolerated, it should be maintained for as long as possible. As for MAD, it consists of reducing carbohydrate intake to 10 to 20g/day, being effective in school-age children and adolescents; it can be an alternative when classic KD is difficult to tolerate in the long term. 1 Finally, LGIT is not recommended for Glu1DS or PDD as it provides inadequate ketosis. 1

 Recent studies recommend KDTs for patients with KCNT1-related developmental and epileptic encephalopathies, considering them the most common and efficacious therapies beyond other ASMs (levetiracetam, phenobarbital, and quinidine). 23

### Contraindications

 The International Ketogenic Diet Study Group does not recommend KDTs for several disorders ( Table 2 ). 1

**Table 2:** International Ketogenic Diet Study Group's contraindications to KDTs

**Absolute**	Carnitine deficiency (primary)
	CPT I or II deficiency
	Carnitine translocase deficiency
	b-oxidation defects
	MCAD
	LCAD
	SCAD
	Long-chain 3-hydroxy acyl-CoA deficiency
	Medium-chain 3-hydroxy acyl-CoA deficiency
	Pyruvate carboxylase deficiency
	Porphyria
**Relative**	Inability to maintain adequate nutrition
	Surgical focus identified by neuroimaging and video-EEG monitoring
	Parent or caregiver's noncompliance
	Propofol concurrent use (risk of propofol infusion syndrome may be higher)

**Abbreviations:** KDT, ketogenic dietary therapy; CPT, carnitine palmitoyltransferase; MCAD, medium-chain acyl dehydrogenase deficiency; LCAD, long-chain acyl dehydrogenase deficiency; SCAD, short-chain acyl dehydrogenase deficiency; EEG, electroencephalography.

 For KDTs, the metabolism shifts from using carbohydrates to lipids as the primary energy source. Hence, before initiating KDTs, patients should be screened for fatty acid transport and oxidation disorders, as well as inborn errors of metabolism that could lead to a severe metabolic crisis. 1

### Recommendations

The Brazilian Child Neurology Association recommends KDTs for children with pharmacoresistant epilepsy who failed the treatment with two or three ASMs in mono- or polytherapy, if they were properly indicated, tolerated, and used in an adequate dosis.

In patients with Glut1DS and PDD, KDTs should be started as soon as the diagnosis is established. In epileptic syndromes (such as Doose, Dravet, infantile spasms, tuberous sclerosis complex, Angelman syndrome, and Ohtahara syndrome) KDTs can be offered earlier following the failure of adequate trials of two tolerated, appropriately chosen, and administered ASMs (whether as monotherapy or in combination) to achieve seizure freedom. Screening for fatty acid transporter diseases and beta-oxidation defects should be performed in patients without a defined etiology before starting the treatment.

## PREDIET EVALUATION

 An initial appointment is scheduled after establishing which patients are eligible for treatment. A multidisciplinary team constituted of at least a child neurologist and dietitian should assess the patient and caregivers. 20

We recommend examining the patient's history before starting the KD to assess the potential effectiveness of the treatment and define the epileptic syndrome. If necessary, the KD team should revise the clinical tests, repeat neuroimaging to exclude possible surgical causes, and include genetic investigation.

### Counseling

 Counseling involves discussing the pros and cons of KDTs, seizure reduction, medication changes or dose reduction, and cognitive expectations with family and caregivers. It is also essential to discuss the potential psychosocial and financial impacts of KDTs with the family and caregivers, 1 as they must understand the treatment requires strict adherence for maximal effectiveness. Counseling should take enough time for family members and caregivers to fully understand the therapy. 20

### Nutritional evaluation

 During the nutritional assessment, the following parameters must be checked: 1

- Baseline weight and height. Calculate ideal weight for stature and body mass index (BMI), then compare with referential curves. For patients with physical limitations, the arm span, when the patient's arms are extensible, knee height, tibial length, or arm length can be used;
- Head circumference in infants;
- Nutrition intake history: 3-day and/or 24 hour food record;
- Food preferences, aversions, allergies, and intolerances;
- Diet formulation: infant, oral, enteral, or a combination thereof;
- Diet selection (classic KD, MCT, MAD, and LGIT);
- Ketogenic ratio; calorie intake; indication of MCT oil; calculation of protein recommendations for age and nutritional status; fluid for weight and age;
- Vitamin and mineral supplementation based on dietary reference intake.

### Laboratory evaluation

 A laboratory evaluation is essential to ensure no pre-existing contraindications or deficiencies before starting KDTs. 7 This evaluation includes: 1

- Complete blood count with platelets;
- Blood glucose;
- Electrolytes, serum bicarbonate, total protein, and calcium;
- Serum, liver, and kidney tests (including albumin, blood urea nitrogen, and creatinine)
- Fasting lipid profile;
- Serum acylcarnitine profile, if economically possible and applicable according to epilepsy etiology;
- Vitamin D level;
- Urinalysis;
- ASMs levels (if applicable).

Additional tests:

- Electroencephalography (EEG);
- Brain 3 tesla (3T) magnetic resonance imaging (MRI);
- Echocardiogram, if there is a history of heart disease;
- Urine organic acids (if diagnosis unclear);
- Serum amino acids (if diagnosis unclear);
- Genetic testing;
- Next-generation sequencing.

### Recommendations

 Laboratory tests should be requested before starting the treatment ( Table 3 ). In regions with limited resources, request at least the mandatory tests. 

**Table 3:** Recommended laboratory tests for treatment initiation

**Mandatory**	Sodium
	Potassium
	Bicarbonate
	Chlorine
	Urea
	Creatinine
	Blood glucose
	Metabolic tests to identify the etiology, especially when there is a suggestive clinical condition and family history
	Complete blood count (especially in regions where anemia is frequent)
	Vitamin D (especially when using a first-generation ASM)
	Lipid profile (highly recommended if there is a family or personal history of hyperlipidemia or cardiovascular risk)
**Recommended**	Liver function (mandatory if the ASM is metabolized by the liver)
	Calcium
	TSH, T4
	Vitamin B12 and folic acid
**Optional**	Free carnitine
	Selenium
	Magnesium
	Phosphorus
	Urine (routine)
	Serum ASM level

**Abbreviations:** ASM, antiseizure medication; T4, thyroxine; TSH, thyroid-stimulating hormone.

## DIET SELECTION

 There are four main KDTs: classic KD, MCT, MAD, and LGIT. The International guidelines recommend choosing the diet according to the family and child's clinical condition and preferences, in addition to perceived efficacy or the center's expertise. 1

 Additionally, children younger than two should start with the classic KD (a formula-based one may be helpful). Both MAD and LGIT are recommended for adolescents, although classic KDs could be used in individual cases, especially with enteral feeding. 1

## INITIATION OF THE DIETARY THERAPY

 The European guidelines on KDT for infants with pharmacoresistant epilepsy recommend that infants (<12 months) should be admitted to the hospital when initiating the KDT. The diet should start at a 1:1 ratio and slowly progress to the classical 3:1 ratio. This ratio can be adjusted according to ketosis levels and tolerance. 7

 The International guidelines state that flexibility is well supported in this initial phase. Fasting may be appropriate when a quicker time to respond is desired. Still, it does not affect the treatment's long-term efficacy, and its side effects are more immediate. 1 The group also recommends starting the KDT in outpatients when appropriate. Most clinics start the MAD and LGIT in outpatients without a fasting period. 1

 Hospitalization could help reduce the child's stress and the need for laboratory tests, reducing the treatment costs. However, it requires monitoring blood glucose and ketones for possible adverse events. 20

 Infants can continue bottle feeding while on KDT. It is also possible to continue using a limited amount of breastmilk combined with a ketogenic 3:1 formula which can be given by bottle or tube. In some cases, breastfeeding is possible after giving the child a controlled amount of a 4:1 ketogenic formula. 7

### Recommendations

Most Brazilian centers have experience with classic KD and MAD. We do not recommend fasting in a hospital setting before starting the transition diet, except in status epilepticus. Typically, children should start the treatment at home. Hospitalization is always indicated for children under 2 years of age; for older patients, it depends on their clinical condition and the service experience.

Flexibility and personalization must be adapted for each patient, according to dietary acceptance and economic conditions. Most centers recommend reducing carbohydrate consumption before starting the diet.

In the first appointment, parents, caregivers, and others involved in preparing the meals for the patient should be present. They should bring a digital scale, materials to measure ketosis (urinary or blood), and a diary to take notes regarding the KDT.

 We recommend the classic KD in the 1:1 or 2:1 proportion for children with full calories from the beginning. Fat is gradually added to the 2:1 or 3:1 ratio in the 2 ^nd^ week. In the 4 ^th^ week, measurements can move on to the 3:1 or 4:1 ratio, when the patient remains with seizures associated with low ketosis. In the absence of adverse effects or food refusal, the diet may be progressed every 1 to 2 weeks to reduce the seizures. On the contrary, the dietitian must adjust the meal plan, manage adverse effects, and evolve only after patient stabilization and adaptation. Treatment should be maintained in the proportion on which the patient had a good clinical response, with good tolerability, and adequate ketosis. 

 In hospitalized children younger than 1 year old who need to start the KDT immediately, we start the KDT in a 1:1 ratio, with the total amount of daily calories evolving to a 2:1 ratio on the 2 ^nd^ day, and to a 3:1 ratio on the 3 ^rd^ day. Breastfeeding infants and children can also be treated with the KDT. 

We also recommend hospitalization for families who need intensive training before starting treatment.

## MONITORING DURING DIET INITIATION

 The European and International guidelines guidelines provide recommendations for monitoring the patients during the initial phase of treatment. 1 7

 Due to the risk of hypoglycemia, blood glucose should be checked twice daily. Furthermore, during the transition to KDT, body ketone levels increase. Monitoring ensures ketones will reach a therapeutic level without causing excess ketosis. Blood ketones can be measured twice daily. 1 7

 If symptoms or signs of hypoglycemia or ketosis occur, such as fatigue, dry mouth, or dizziness, the measurements should be more frequent. Proper treatment should be initiated when necessary. 7 20

 At this stage, it is also vital to monitor adverse events, such as gastrointestinal reactions like vomiting, nausea, diarrhea, constipation, and abdominal discomfort. 7

### Recommendations

In the first 3 months, urinary or blood ketosis should be performed twice daily, when possible, before meals. Putting a cotton ball inside for children who wear diapers prevents urine absorption. It is essential to keep the same time of day to perform the measurements to avoid interference with the circadian cycle and hormonal changes.

 Blood glucose should be measured twice a day in the 1 ^st^ month. Parents/caregivers should be advised to observe clinical symptoms of hypoglycemia and hyperketosis. 

## CONCOMITANT PHARMACOLOGIC TREATMENTS

 There is little evidence of beneficial interactions between KDTs and ASMs. There is also no particular ASM that should be avoided. The International Ketogenic Study Group recommends reducing ASMs after 1 month of successful KDT. However, they advise caution when reducing phenobarbital or benzodiazepines. 1

 One study found that the mean concentrations of ASMs such as carbamazepine, clobazam, valproate, lacosamide, lamotrigine, and topiramate were significantly reduced in adults with pharmacoresistant epilepsy following a MAD. 24 However, an open clinical study of 51 children with pharmacoresistant epilepsy found that the KD did not significantly change the plasma concentration of commonly used ASMs. Therefore, adjusting for drug doses due to pharmacokinetic interactions is unnecessary. 25

### Recommendations

Since the KDT effectiveness is evaluated after three months of treatment, it is essential that, within this period, changes in drug doses are kept to a minimum in order not to interfere with the KDT therapeutic evaluation. Typically, ASMs reduction starts after the first month of diet as soon as some success with the diet is observed. It is important to ensure that the diet is well tolerated and it is not causing exacerbation of crises.

## ENERGY REQUIREMENTS

 According to the European guidelines, the energy requirements of infants with epilepsy should be based on food diary records and compared with the recommended dietary allowances (RDA) by age, gender, and recent growth. 7

 A recent decline in the growth curve indicates a need for additional energy. It is also essential to determine the ideal weight/age or weight/height to ensure catch-up growth and avoid excessive weight gain. 7

 The energy requirements must be adjusted frequently according to growth curve evaluations, activity levels, and illness development. 7

### Nutrients

 The European guidelines provide recommendations for fat, protein, carbohydrate, and fluid intake for infants. 7

 Protein intake should be based on RDA, and adjustments may be required based on the child's weight. 7 Carbohydrate intake can be calculated based on energy, protein, and fat requirements. This intake is divided throughout the day to prevent adverse events like hypoglycemia or excessive ketosis. 7 Also, introducing solid foods when age and developmentally appropriate increases the amount of fiber intake, preventing constipation. 7

 Fluid restriction is not recommended since it can cause kidney stones and dehydration; its intake should follow the RDA by age and gender. 7

### Recommendations

 The European guidelines are specific for infants. Therefore, there is a need to expand the recommendations to other age groups, including newborns. The child dietitian will calculate the adequate diet for each patient according to their age, nutritional status, and route of administration. The individualized diet plan should contain varied meals according to caloric needs and the appropriate proportion of macronutrients. 26

 The 2002/2005 dietary reference intakes (DRI) are used to estimate the basal metabolic rate (BMR) according to the patient's age, weight, height, and activity level. 26 The estimated energy requirements (EER) are calculated via energy expenditure (EE), BMR, and physical activity (PA) coefficient. 26

 Some authors calculate the energy requirements between 80 and 90% of the DRI for children of 3 or more years of age, but this is not a consensus. 27 The energy requirements should be individualized and can be altered anytime during the treatment. Even in patients younger than 3 years old with significant movement restrictions, adjusting to a higher caloric intake may be necessary since they tend to gain weight quickly. 27

 Careful monitoring allows individualized adjustments. In general, the protein intake is 1g/kg/day of protein for children older than 1 year old and 1.2g to 1.5g/kg/day for children younger than that due to the accelerated growth, which increases protein needs. 28

 The amount of liquid should be calculated according to the basal needs. 29 We suggest using the Holliday-Segar formula to ensure the correct minimum daily intake. Fluid restriction is not recommended. 29

 In Brazil, four meals a day are usually offered (breakfast, lunch, afternoon snack, and dinner) but this number can vary from three to five meals a day, depending on the needs and habits of each patient and family. Children under 1 year old in KDT with a 4:1 industrialized formula or blenderized food could receive up to six meals daily. 30 For patients who feed via tube or gastrostomy, it is essential to adjust the menu to a more liquid/pasty consistency to avoid obstructions in the route. 30

## MAINTENANCE

 We believe follow-ups should be personalized for each patient. Children on KDT must have regular follow-ups with the multidisciplinary KD team. According to the international consensus group, these follow-up visits should happen every 3 months in the 1 ^st^ year. After that, the visits can occur every 6 months. Children at high risk for nutritional deficiency should be seen more often. 1

 Follow-up visits should include nutritional assessment, medical evaluation, and laboratory tests. 1 The nutritional assessment must be carried out by a registered dietitian and should include height, weight, growth velocity, BMI, KDT prescription appropriateness review, dietary supplementation review, diet compliance, and necessary adjustments. 1

 The medical evaluation by a pediatric neurologist should cover the KDT's efficacy and side effects, considerations about ASM reduction or diet discontinuation, and ensure proper supplementation. 1

In the first few months of KD, several adjustments are necessary to maintain ketosis and adjust acceptance. The constant presence of the child dietitian is important to support and ensure adherence.

 Laboratory tests should include complete blood count with platelets, electrolytes (serum bicarbonate, total protein, calcium), serum liver and kidney profile, vitamin D, fasting lipid profile, free and total carnitine (if receiving ASMs such as valproate), urinalysis, anticonvulsant drug levels, and EEG (at KDT discontinuation consideration). Further, optional, tests are serum beta-hydroxybutyrate (BOH) levels, selenium, urine calcium and creatinine, zinc and copper levels, renal ultrasound, electrocardiogram (ECG), and bone mineral density with a dual x-ray absorptiometry (DEXA) scan. 1

### Dietary supplementation

 Supplementation is essential for people on KDTs due to the limited intake/exclusion of fruits, vegetables, enriched grains, and foods containing calcium and vitamin B. Evidence suggests that children with epilepsy may have decreased vitamin D levels due to the interaction with certain ASMs. 31

 The International Group's consensus recommends that all children receiving KDTs take calcium and vitamin D supplements. 1 Exposure to the sun should also be encouraged. 20

 To ensure adequate supplementation, micronutrients should be individually calculated based on the RDA for the child's age, gender, and weight. 7

### Recommendations

In Brazil, there are no specific food supplements to meet the micronutrient needs of individuals undergoing KDTs. The choice between commercial supplements with a multivitamin formula or a compound prescription will depend on the individual needs.

Among the commercial formulas, we recommend choosing those with a minimal amount of carbohydrates. Alternatively, a compound prescription of the vitamins and minerals should be offered according to the patient's age and nutritional needs. Calcium and vitamin D can be administered separately through calcium carbonate tablets and commercial vitamin D supplements.

 Patients using valproic acid may develop hypocarnitinemia and hypothyroidism, and require L-carnitine supplementation. 32 Carnitine dosing is not routinely performed by all centers. In our center, we only perform carnitine supplementation in case of documented deficiency or, eventually, in infants with clinical symptoms of possible deficiency, fatigue, difficulty in reaching adequate ketosis values, and use of drugs that reduce the availability of free carnitine. 

Patients using exclusively commercial ketogenic formulas are probably under the dietary recommendations. The commercial formula's label will provide information in order to verify if supplementation is needed. During follow-up, it is essential to frequently assess the patient's clinical nutritional status to ensure fine adjustments of the diet and supplementation.

 During follow-up, in regions with limited resources, it is important to request at least the mandatory tests ( Table 4 ). 33 Other analyses that can be helpful include thyroid-stimulating hormone (TSH), thyroxine (T4), and vitamin B12 and folic acid levels in the blood. 

**Table 4:** Recommended laboratory tests during treatment follow-up

**Mandatory**	Sodium, potassium, bicarbonate (CO2), chlorine, urea, creatinine, blood glucose, lipid profile, and urinalysis.
**Recommended**	Liver function (if using ASMs metabolized in the liver), vitamin D, complete blood count, calcium, free carnitine (highly recommended if using ASMs such as valproate)
**Optional but not required**	Selenium, magnesium, phosphorus, serum ASM levels, renal ultrasound, Calcium/Creatinine ratio in urine, and bone densitometry.

**Abbreviation:** ASM, antiseizure medication.

## ADVERSE EVENTS

 Adverse events may occur in children on KDTs, especially during the initial phase, and should be monitored to ensure tolerability. These symptoms are usually mild and easily manageable with dietary manipulation and medications. 6 27 28 29

 The most common adverse events during KD initiation are gastrointestinal reactions, transient food refusal, lethargy, and hypoglycemia. Constipation, vomiting, and acidosis may also occur. 34

 Around 3.2 to 4.6% of children report hyperlipidemia, hypercholesterolemia, or hypertriglyceridemia during KDT. 35 Strategies to prevent KDT-induced hyperlipidemia include increasing MCT and olive oil consumption, supplementing with omega-3 fatty acid or carnitine, and decreasing trans-fat, saturated fat, and cholesterol intake. 1 36 37 Evidence shows these abnormalities are transient, resolving after treatment discontinuation. 38

 About 6% of children on KDT report kidney stones. In those treated for more than 6 years, the prevalence was of 25%. 39 However, it rarely requires lithotripsy or leads to KDT discontinuation. 39 Furth et al. (2000) suggested a potential benefit of urine alkalinization with potassium citrate for individuals with a urine calcium-creatinine ratio greater than 0.2 mg/mg. 40 Potassium citrate solubilizes calcium, decreasing free calcium available for crystallization. It also increases urinary pH, allowing the dissolution of uric acid crystals. 41 42 According to Kossof et al; (2002), patients using carbonic anhydrase inhibitors (such as topiramate) and with a positive family history of kidney stones are at greater risk and should be treated with potassium citrate. 43

 The most frequently described cardiac alterations during KDT are cardiomyopathy due to selenium deficiency, complications of increased QT interval also related to selenium deficiency, low bicarbonate levels, high β-hydroxybutyrate levels, and carnitine deficiency. 44 45 46 47

 There is no consensus in the literature regarding the effects of KDTs on children's growth. A systematic review included ten studies that evaluated growth in children with pharmacoresistant epilepsy receiving the classic or MCT diet. There were two studies that found that KDT had a positive effect on growth. In comparison, six studies concluded that the diets had a negative effect on growth. Finally, two studies showed no significant difference in children's growth while on KDT. 35 Nevertheless, the international guidelines recommend assessing anthropometric data and comparing it to growth patterns on each visit. 20

 Other possible complications include hypoglycemia, dehydration, nutritional deficiencies, acid reflux, hunger, weight loss, and hepatic dysfunction. 7 35 44 46 47 48

 Long-term adverse effects usually appear after the initial 3 months of KDT and include hyperlipidemia, gastrointestinal alterations, kidney stone, growth deficiency, bone and cardiac alterations, and vitamin and mineral deficiencies. 34 Overall, the risk of serious adverse events is low; most are transient and do not require KDT discontinuation. 1 7

### Recommendations

 Adverse events may occur during the initial phase of treatment with KDT, especially during the 1 ^st^ month, but they are usually mild and easily manageable. These initial complications can be stressful for the family and lead to discontinuation. Therefore, we recommend discussing possible adverse events in the first appointments with the family. The caregivers should learn how to recognize them and provide early treatment; then, patients must be referred to a multi-professional team. 35 49

Hypoglycemia and acidosis may occur in the initial phase of the treatment. These adverse events are more common and intense in younger children and when fasting precedes the KDT. They can also be present during maintenance under metabolic stress, such as fever and infection. Dehydration, lethargy, gastrointestinal alterations, and appetite loss may also occur.

 To avoid early treatment discontinuation, it is crucial to reinforce that the initial transitory difficulties do not interfere with the diet's effectiveness. 35 49

## DISCONTINUATION

 The main goal of KDTs in pharmacoresistant epilepsy is to reduce seizures and ASMs intake. 7 As stated by the International Group's consensus, the KDT should be discontinued after 3 months of unsuccessful treatment. 1

 The Ketogenic Diet Working Group of the Argentine Society of Pediatric Neurology allows for a longer interval to consider treatment failure (6 months). The KDT team must formally evaluate the therapy's effectiveness and discuss the outcomes with the family. This evaluation should consider aspects such as tolerance and adherence. 20

 The therapy typically continues for at least 2 years in children who have achieved seizure control. 18 There is evidence that seizure control can be maintained after returning to a regular diet, for children who have had a positive response to a KDT. 7 38

 The discontinuation should follow a gradual wean over 1 to 3 months unless there's an urgent need. The KDT ratio is slowly reduced monthly; 7 in seizure-free children, routine EEG can be valuable. 1

 The Ketogenic Diet Working Group of the Argentine Society of Pediatric Neurology recommends decreasing 1 point in the ketogenic ratio every 2 to 3 weeks, 2 to 3 years after initiation. They also recommend extending it for a longer period if seizure reduction with the diet is over 90%, if the number of seizures increases during reduction of the ketogenic ratio, as well as in patients with specific syndromes, such as tuberous sclerosis complex or Dravet syndrome. 20

 The discontinuation timing and method can be individualized according to the patients' response. A shorter diet duration, 6 months, for instance, may be adequate for patients with infantile spasms and status epilepticus. 1

### Recommendations

There is no consensus regarding the ideal way to interrupt the KDT and how long it should take. The KD team must come to a decision alongside parents/caregivers.

 The duration of KDT depends on the percentage of seizure reduction. Patients with a ≥ 50% decrease in seizure frequency should continue the treatment for2 to 3 years. When patients achieve > 90% seizure reduction, adverse effects are negligible, and the chance of recurrence of seizures is high—like in tuberous sclerosis and Dravet syndrome—KDT can be maintained indefinitely, controlling for adverse events, and evaluating the risk–benefit ratio that a high-fat diet can cause in the long term. Periods of 12 years of treatment have been described. 48

 Conversely, KDTs can be maintained indefinitely in certain conditions, such as for Glut-1DS and PDD patients. 1 This period of time can be particularly delicate for parents and caregivers, who may need additional support. 6

In conclusion, the KD is a safe and effective therapy for managing pharmacoresistant epilepsy in children. The Brazilian Child Neurology Society recommends its use for child patients who failed the treatment with two or three ASMs.

The prediet evaluation is critical, and involves establishing a multidisciplinary team to assess the patients and their caregivers' specific needs.

It is also essential to monitor for any sign of an adverse event during the dietary treatment. In general, adverse events are mild and easily manageable.

The main goal of the KD treatment is to reduce seizures and ASMs intake. Discontinuation must be carefully planned. The diet can be maintained independently under certain conditions.

Hopefully, these recommendations will help spread the use of the KD in a socially and culturally diverse country such as Brazil.
